# P-52. Hazard before the hardware? Bloodstream infections prior to implantation of durable left ventricular assist devices (LVAD)

**DOI:** 10.1093/ofid/ofaf695.281

**Published:** 2026-01-11

**Authors:** Patrick C Tam, Manuela Carugati, Arthur W Baker, Sana Arif, Madeleine R Heldman, Eileen K Maziarz, Cameron Wolfe, Ilan Schwartz, Bin Ni, Brennan Collis, Michael E Yarrington, Sonya Kothadia

**Affiliations:** Duke University School of Medicine, Durham, NC; Duke University, Durham, North Carolina; Duke University School of Medicine, Durham, NC; Duke University, Durham, North Carolina; Duke University, Durham, North Carolina; Duke University Medical Center, Durham, NC; Duke University Hospital, Durham, North Carolina; Duke University, Durham, North Carolina; Duke University Medical Center, Durham, NC; Duke University Hospital, Durham, North Carolina; Duke University Health System, Durham, North Carolina; Duke University, Durham, North Carolina

## Abstract

**Background:**

Implantable mechanical circulatory support (MCS), such as left ventricular assist devices (LVADs), can improve survival in patients with end-stage heart failure. While post-implant complications including MCS-specific infections are well recognized, the outcomes of LVAD recipients with bloodstream infection (BSI) prior to device implantation have not been systemically studied.

**Figure:**
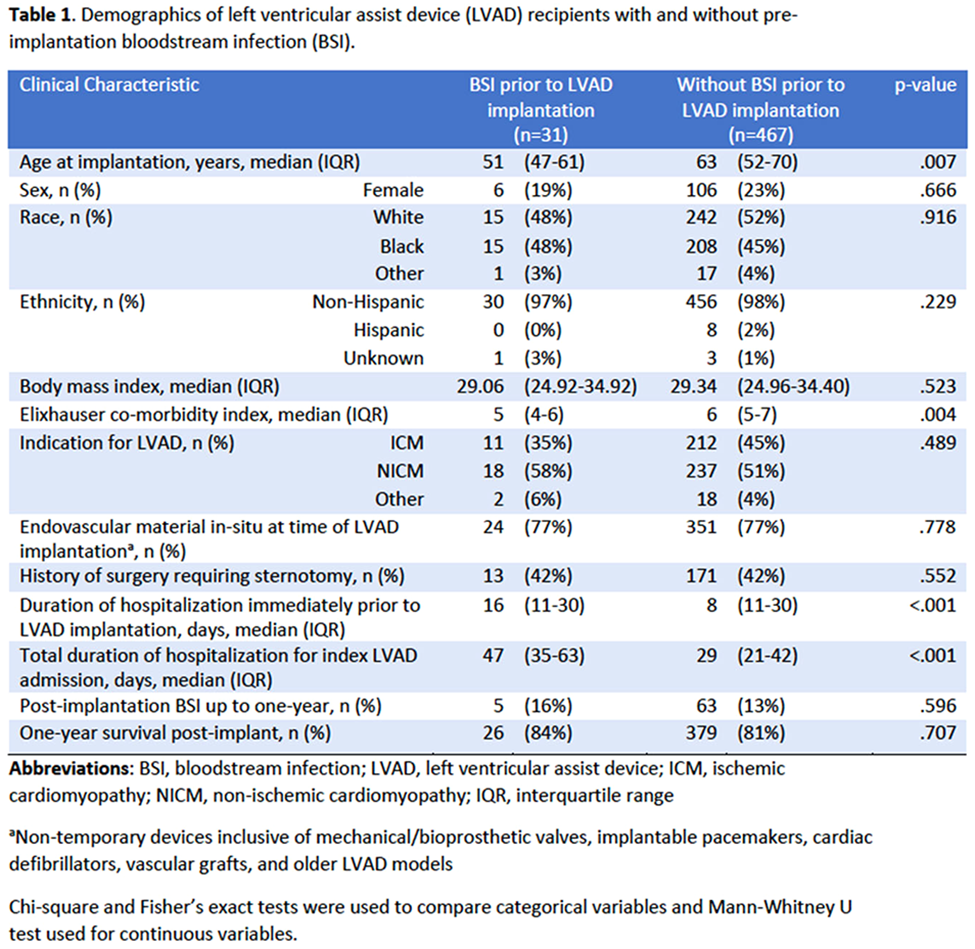

**Figure:**
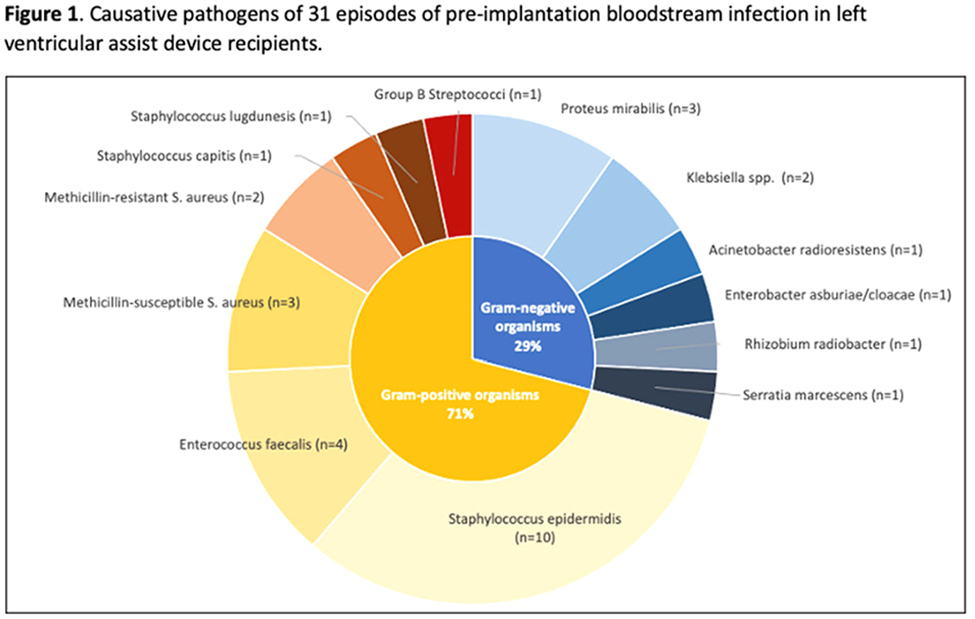

**Methods:**

We performed a single center retrospective study of all consecutive persons who received a Heartmate 3 (HM3) LVAD implanted at our institution between 1/1/17-12/31/23. We compared recipients with and without clinically significant BSI in the three months preceding device implantation. For these two groups, we also compared demographics, clinical characteristics, and outcomes, including post-implant BSI and one-year survival.

**Figure:**
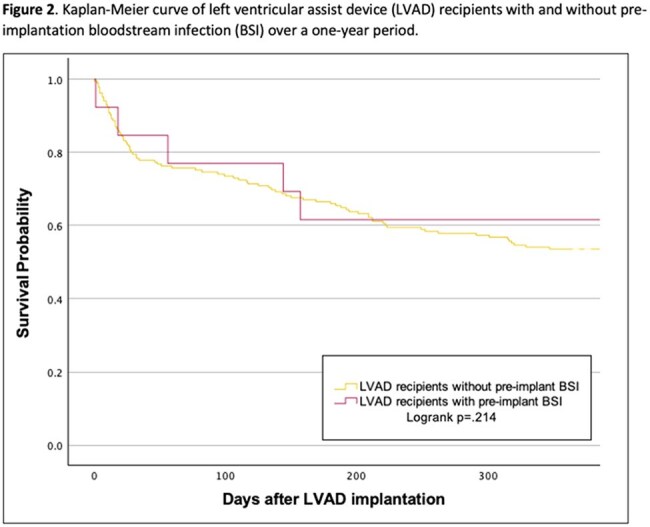

**Results:**

Over the seven-year period, 498 patients received a HM3, of whom 31 (6%) had a pre-implant BSI (Table 1). The median time from pre-implant BSI to LVAD surgery was 13 days (interquartile range 6-18). The most common pathogens were Gram-positive organisms (22; 71%; Figure 1). Patients with pre-implant BSI were younger (p=.007), had lower Elixhauser co-morbidity indices (p=.004), and were in hospital longer immediately prior to LVAD surgery (p< .001) compared to patients without pre-implant BSI. Cumulative incidence of one-year post-implant BSI was similar in those with and without pre-implant BSI (16% v. 13%; p=.596). Of 31 patients with pre-implant BSI, 11 (35%) developed post-implant MCS-specific infection in the first year (n=9 percutaneous lead infections, n=1 device-specific BSI, n=1 infection of external surfaces of implant). Only one (3%) MCS-specific infection was attributed to the same pathogen (*S. aureus*) isolated in blood culture prior to implant. Patients with pre-implant BSI had longer index hospitalizations after LVAD implantation (47 v. 29 days; p< .001) but similar one-year survival compared to patients without pre-implant BSI (84% v. 81%; p=.707; Figure 2).

**Conclusion:**

Pre-implant BSI in LVAD recipients was not associated with post-implant BSI. One-year survival was similar between LVAD recipients with and without pre-implant BSI. The risk factors, management, and outcomes of pre-implant BSI in LVAD recipients warrant further study.

**Disclosures:**

Madeleine R. Heldman, MD, MS, Karius, Inc: Advisor/Consultant Cameron Wolfe, MBBS, Brio-VAD: Advisor/Consultant

